# Collision with Gondwana or with Baltica? Ordovician magmatic arc volcanism in the Marmarosh Massif (Eastern Carpathians, Ukraine)

**DOI:** 10.1007/s00531-022-02228-8

**Published:** 2022-07-16

**Authors:** Aleksandra Gawęda, Krzysztof Szopa, Jan Golonka, David Chew, Leonid Stepanyuk, Volodymir Belskyy, Anna Waśkowska, Laurynas Siliauskas, Foteini Drakou

**Affiliations:** 1 Institute of Earth Sciences, University of Silesia in Katowice, Będzińska 60, 41-200 Sosnowiec, Poland; 2 Faculty of Geology, Geophysics and Environmental Protection, AGH University of Science and Technology, al. A. Mickiewicza 30, 30-059 Kraków, Poland; 3 Department of Geology, School of Natural Sciences, Trinity College Dublin, Dublin 2, Ireland; 4 M.P. Semenenko Institute of Mineralogy, Geochemistry and Ore Formation of the National Academy of Sciences of Ukraine, Palladina av. 34, Kyiv, 142 Ukraine

**Keywords:** Ukraine, East Carpathians, Tornquist ocean, Caledonian volcanism

## Abstract

The pre-Alpine Marmarosh Massif is a tectonically complex unit of the crystalline basement within the Eastern Outer Carpathians. In the eastern (Ukrainian) segment of this massif, two units have been identified—the Bilyi Potok Nappe and the Dilove Nappe. Petrological investigations coupled with zircon U–Pb dating were performed on metavolcanic rocks (porphyroids) and their phyllite host rocks, sampled from three locations within the Dilove Nappe. The geochemical characteristics of the meta-rhyodacite porphyroids revealed a volcanic arc affinity of the protolith, with U–Pb zircon ages of 452.8 ± 1.5 Ma and 461.5 ± 1.6 Ma and zircon saturation temperatures in the range of 823–892 °C. The phyllite host rocks (meta-tuff) yield a U–Pb zircon maximum estimate for the eruption age at 584.7 ± 2.9 Ma. Peak amphibolite-facies metamorphism (M1) was estimated at the pressure of 600–900 MPa with a temperature range of 560–600 °C. Retrogression (M2), possibly related to Alpine nappe stacking and shearing, is assumed to have taken place at 200–250 MPa and 384–222 °C. The volcanic arc is interpreted as an early Caledonian arc that was subsequently accreted to the margin of Baltica during the closure of the easternmost Tornquist Ocean rather than Cenerian (early Paleozoic) orogenic events on the margin of East Gondwana.

**Supplementary Information:**

The online version contains supplementary material available at 10.1007/s00531-022-02228-8.

## Introduction

**Fig. 1:**
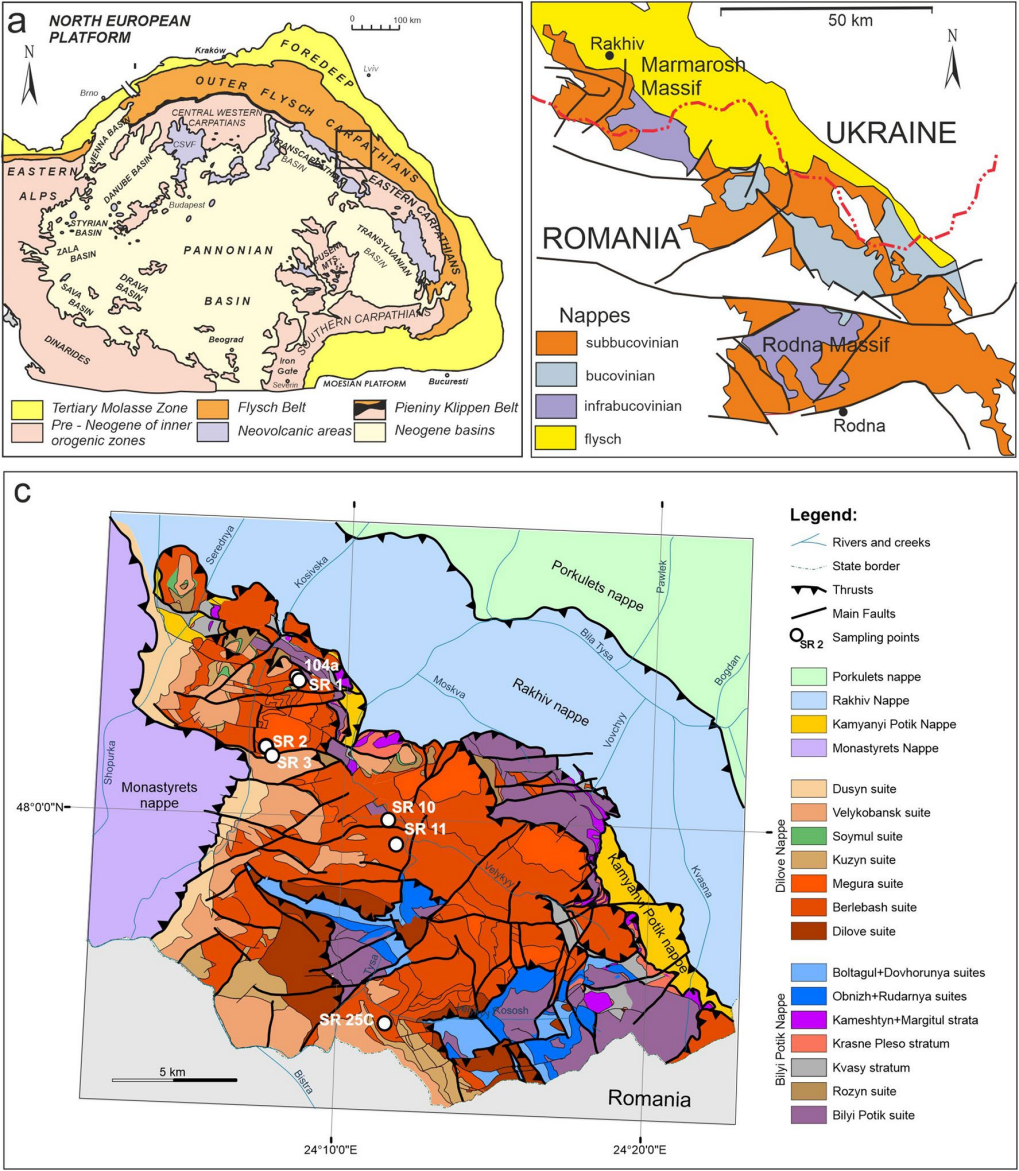
**a** Simplified geological map of the Carpathian Chain. **b** The location of the Marmarosh massif and its relationship to the Rodna Horst (modified after Balintoni and Balica 2013). **c** Geological map of the Ukrainian part of the Marmarosh Massif with the sampling localities indicated (modified after Matskiv et al. 2006)

The arcuate Carpathian mountain belt in Central Europe, extends for more than 1300 km from Vienna Basin to the Iron Gate gorge on the Danube (Fig. 1a). Traditionally, the Carpathians are divided into the internides, deformed mainly in the Mesozoic and containing crystalline cores of pre-Alpine (mostly Variscan) basement (Schmidt et al. 2008; Golonka et al. 2021a and references therein), and the externides that were deformed in the Cenozoic (Golonka et al. 2006; Schmidt et al. 2008). Geographically, the Carpathian orogen is divided into the Western, Eastern and Southern Carpathians. The externides consist of a complex nappe system, that developed during Cretaceous to Neogene times as a result of the collision of several microplates with the consolidated European Plate. The nappes generally verge towards the outer part of the arc (Golonka et al. 2021a and references therein). The polyphase-deformed basement on which the Outer Carpathian basins developed is termed the Protocarpathian Massif or simply the Protocarpathians. In the Western Outer Carpathians (Czech Republic, Slovakia and Poland), the Protocarpathian basement is covered by several kilometres of marine Mesozoic to Cenozoic clastic sediments of the Carpathian basins. The nature of the basement is therefore known only from olistoliths and exotic pebbles redeposited in these clastic sequences (e.g., Poprawa et al. 2006; Gawęda et al. 2019 and references therein; Gawęda et al. 2021; Golonka et al. 2021b).

However, the crystalline basement of the Protocarpathians is exposed in the Eastern Outer Carpathians in Ukraine, in the Marmarosh Massif (Fig. 1b). This massif is thus key to understanding the tectonic affinity of this eastern part of the Protocarpathian basement. It is composed of pre-Alpine crystalline rocks, mostly metamorphic basement, overlain by sedimentary rocks that were thrust northwards during the Alpine closure of the Carpathian branch of Tethys. The basement remnants likely represent a fragment of the North European Platform that rifted away during the Jurassic opening of the Protosilesian-Severin-Moldavidic Basin (Golonka et al. 2021a and references therein). The Ukrainian Outer Carpathian crystalline rocks extend southeastwards into the Eastern Carpathians of Romania, forming the Median Dacides, which are in turn divided into the Bucovinian, Sub-Bucovinian and Infra-Bucovinian nappes. Recent petrological and isotope dating studies on the Romanian crystalline basement (Balintoni and Balica 2013; Balintoni et al. 2014) enable the correlation of these rocks with their Ukrainian equivalents. The correlation of the Marmarosh Massif with the Protocarpathian basement in the Western Outer Carpathians is quite speculative (see Ślączka et al. 2006). However, several studies (e.g. Bonova et al. 2019; Oszczypko et al. 2005, 2020) argue for the Marmarosh Massif as a source for clastic material in the various flysch deposits of eastern Slovakia and even in the central part of the Polish Outer Carpathians.

This study combines geochemical and petrological information, U–Pb LA-ICP-MS zircon dating and P–T constraints on meta-igneous rocks from three localities within the Dilove Nappe of the Marmarosh Massif. These data are then used to test correlations with the Romanian segment of the Eastern Carpathians, and to interpret the paleotectonic setting of protolith rocks with respect to the evolution of the Baltica and Gondwana margins during the early Paleozoic.

## Geological setting and sampling

The crystalline Marmarosh Massif is situated northeast of the Pieniny Klippen Belt and the Magura Nappe (Ślączka et al. 2006). The Pieniny Klippen Belt constitutes the boundary between the Carpathian internides and externides, while the Magura Nappe is the innermost Outer Carpathians unit in the Western Outer Carpathians and the adjacent segments of the Eastern Outer Carpathians in Ukraine and Romania (Fig. 1a). Northeast of the Marmarosh Massif (Fig. 1b) is a broad belt of nappes which are from southwest to northeast: the Kaminnyi Potik, Rachiv, Porkulets, Dukla, Chornohora, Krosno, Skole-Skyba and Boryslav-Pokuttya nappes. These nappes can be correlated with Outer Dacides and Moldavides in Romania (Ślączka et al. 2006). They are composed of clastic sedimentary rocks that were originally deposited in the Protosilesian-Severin-Moldavidic Basin, a continuation of the Protosilesian Basin of the Western Carpathians, which developed within the North European Platform as a rift and/or back-arc basin. Its basement is believed to represent the attenuated crust of the North European Platform (the Ceahlau-Severin Ocean, see Schmidt et al. 2008; Matenco et al. 2010).

The Ukrainian part of Marmarosh Massif consists of two units (nappes): the Bilyi Potik Nappe and the Dilove Nappe (Fig. 1c). Both nappes can be broadly correlated with Bucovinian Nappe and Sub-Bucovinian Nappe in Romania, including the Rodna Horst, with which it shares a similar structural configuration (Balintoni et al. 2009; Culshaw et al. 2012).

The Bilyi Potik Nappe (Sub-Bucovinian Nappe) is composed mostly of amphibolite-facies gneisses and schists, locally garnet- and staurolite-bearing, intercalated with amphibolites (*Bilyi Potik Suite*) and overthrust by late Paleozoic greenschist-facies phyllites with marble and dolomitic marble lenses (*Rozin Suite*).

The greenschist-facies Dilove Nappe (Bucovinian Nappe) is comprised of the *Dilove Suite* of porphyroblastic schists, mica-bearing porphyroids (sometimes with relics of garnet), layers of marble and lenses of amphibolites (total thickness of more than 1000 m), the *Berlebash Suite* of calcareous schists and quartzites and metavolcanics of rhyolite-dacite composition (total thickness 1100–1400 m) and the *Megura Suite* consisting of quartzites, quartzite shales, intercalated with quartz-chlorite-sericite shales and with a thickness of 400–460 m. All these components are covered by weakly metamorphosed rocks of the *Cuzyn Suite,* which is comprised of phyllites, carbonaceous shales, quartzites, dolomites and marbles with jasper lenses with a total thickness of 360–550 m, and non-metamorphosed Cretaceous-Paleogene sediments.

For the purposes of our study, we sampled three rock types from *Berlebash suite* of the Dilove Nappe, all showing the same metamorphic grade and interpreted as metavolcanics/metatuffs (Fig. 1c). These samples are: (1) a gneissic porphyroid, interpreted as a meta-rhyolite (archive sample UM-104a from the L. Stepanyuk collection and three new samples (SR1, SR2 and SR3) from an outcrop in Kostylivka village sampled on the right side of the Tysa river (48°09′10″N and 24°11′12″E), (2) a gneissic porphyroid that forms a stock-like body (Boyko et al., 1970) (two samples: SR10, SR11) and sampled near the road at Rakhiv-Kosivska Polana (47°59′29″N and 24°06′11″E) and (3) a strongly folded medium-grained mica schist (phyllite, sample SR25C), interpreted as meta-tuff (Stepanyuk 1986) and sampled from an outcrop located 1.5 km south of the railway bridge over the Tysa River in Kostylivka village, on the west side of the Rakhiv-Dilove highway (47°59′17.6″N and 24°11′53.5″E) (Fig. 1c).

## Analytical techniques

### Microscopy

Petrographic analyses of thin sections were undertaken at the Faculty of Earth Sciences at the University of Silesia using Olympus BX-51 and SX-10 microscopes to constrain textural and microstructural relationships and to determine the presence of zircon.

### Electron probe micro-analyses (EPMA)

Microprobe analyses of the main rock-forming and accessory minerals were carried out at the Inter-Institutional Laboratory of Microanalyses of Minerals and Synthetic Substances, Warsaw, using a CAMECA SX-100 electron microprobe. The analytical conditions employed an accelerating voltage of 15 kV, a beam current of 20 nA, counting times of 4 s for peak and background and a beam diameter of 1–5 μm. Reference materials, analytical lines, diffracting crystals, mean detection limits (in wt%) and uncertainties were as follows: rutile—Ti (Kα, PET, 0.03, 0.05), diopside—Mg (Kα, TAP, 0.02, 0.11), Si—(Kα, TAP, 0.02, 0.21), Ca—(Kα, PET, 0.03, 0.16), orthoclase—Al (Kα, TAP, 0.02, 0.08), and K (Kα, PET, 0.03, 0.02), albite—Na (Kα, TAP, 0.01, 0.08), hematite—Fe (Kα, LIF, 0.09, 0.47), rhodonite—Mn (Kα, LIF, 0.03, 0.10), phlogophite—F (Kα, TAP, 0.04, 0.32), tugtupite—Cl (Kα, PET, 0.02, 0.04), Cr_2_O_3_—Cr (Kα, PET, 0.04, 0.01), ZirconED2—Zr (Lα, PET, 0.01, 0.01), Nb_2_O_3_-MAC—Nb (Lα, PET, 0.09, 0.01), V_2_O_5_—V (Kα, LIF, 0.02, 0.01), YPO_4_—Y (Lα, TAP, 0.05, 0.05), CeP_5_O_14_—Ce (Lα, LPET, 0.09, 0.02), NdGaO_3_—Nd (Lβ, LIF, 0.31, 0.24), ThO_2_—Th (Mα, LPET, 0.09, 0.09), UO_2_—U (Mα, LPET, 0.16, 0.13).

### Whole-rock chemical analyses

Whole-rock analyses were undertaken by X-Ray fluorescence (XRF) for major and large ion lithophile trace elements (LILE), and by fusion and ICP-MS for high field strength elements (HFSE) and rare earth elements (REE) at Bureau Veritas Minerals (Canada). Preparation involved lithium borate fusion and dilute digestions for XRF and lithium borate decomposition or *aqua regia* digestion for ICP-MS. LOI was determined at 1000 °C. Uncertainties for most of the major elements are 0.01%, except for SiO_2_ which is 0.1%. REE were normalized to C1 chondrite (McDonough and Sun 1995).

### Mineral separation and imaging

Zircon crystals were separated using standard density separation techniques (crushing, sieving, washing and panning). This separation was carried out at the Institute of Geological Sciences at the Polish Academy of Sciences, Kraków, Poland. The zircons were hand-picked under a binocular microscope, cast in 25 mm diameter epoxy resin mounts, and then ground and polished to expose the grain interiors. Internal mineral textures were then imaged using back-scattered electron (BSE) and cathodoluminescence (CL) detectors on a FET Philips 30 scanning electron microscope with a 15 kV accelerating voltage and a beam current of 1 nA at the Faculty of Natural Sciences, University of Silesia in Katowice, Poland.

### Zircon LA-ICP-MS U–Pb dating

LA-ICPMS U–Pb age data were acquired using a Photon Machines Analyte Excite 193 nm ArF excimer laser-ablation system with a HelEx 2-volume ablation cell coupled to an Agilent 7900 ICPMS at the Department of Geology, Trinity College Dublin. The instruments were tuned using NIST612 standard glass to yield Th/U ratios of unity and low oxide production rates (ThO^+^/Th^+^ typically < 0.15%). A repetition rate of 11 Hz and a circular spot of 24 μm were employed. A quantity of 0.4 l min^−1^ He carrier gas was fed into the laser cell, and the aerosol was subsequently mixed with 0.6 l min^−1^ Ar make-up gas and 11 ml min^−1^ N_2_. Each analysis comprised 27 s of ablation and 12 s washout, the latter portions of which were used for the baseline measurement. For zircon, eleven isotopes (^49^Ti, ^91^Zr, ^175^Lu, ^202^Hg, ^204^Pb, ^206^Pb, ^207^Pb, ^208^Pb, ^232^Th, ^235^U and ^238^U) were acquired during each analysis. The data reduction of raw U-Th-Pb isotopic data was undertaken using the freeware IOLITE package (Paton et al. 2011), using the “VizualAge” data reduction scheme (Petrus and Kamber 2012). Conventional sample-standard bracketing was then applied to account for both downhole fractionation and long-term drift in isotopic or elemental ratios by normalising all ratios to those of the U-Th-Pb reference materials. Final age calculations were made using the Isoplot add-in for Excel (Ludwig 2012). The primary U–Pb zircon calibration standard was 91,500 zircon (^206^Pb-^238^U age of 1065.4 ± 0.6 Ma; Wiedenbeck et al. 1995; Wiedenbeck et al. 2004) and the secondary standards were Plešovice zircon (^206^Pb-^238^U age of 337.13 ± 0.37 Ma; Sláma et al. 2008) which yielded a concordia age of 340.69 ± 0.99 Ma, and WRS 1348 zircon (^206^Pb-^238^U age of 526.26 ± 0.70; Pointon et al. 2012) which yielded a concordia age of 525.2 ± 2.5 Ma.

## Results

### Petrography and mineral chemistry

**Fig. 2:**
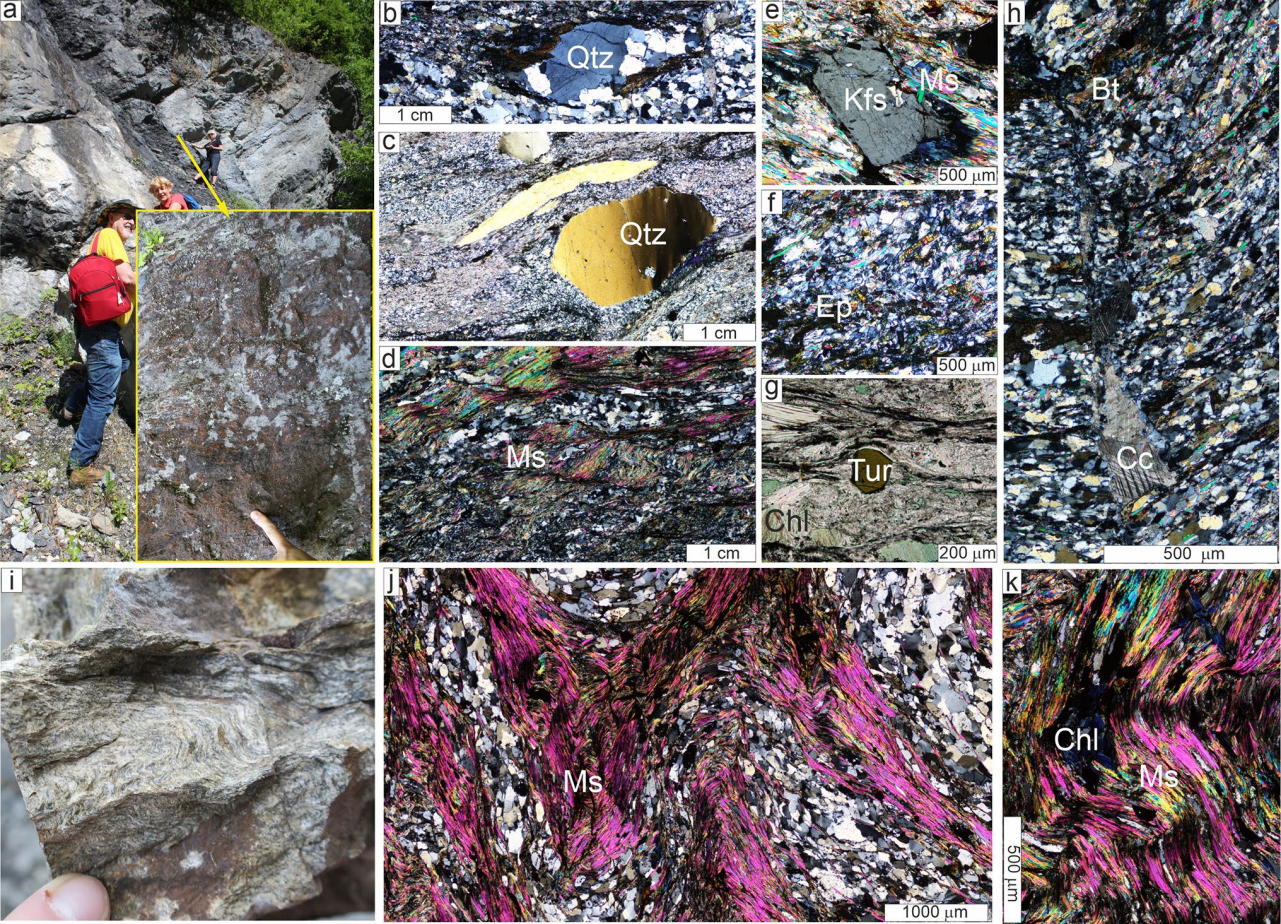
Textures and microtextures of metavolcanic rock from the Marmarosh Massif. **a** field photograph showing the sampling locality for U104a and SR1 samples (J.Golonka, A. Gawęda and A.Waśkowska as the scales) with insert of the rock surface; **b** and **c** deformed quartz (Qtz) porphycrysts within a fine-grained matrix in samples 104a (**b**) and SR10 (**c**) (crossed polars); **d** deformed muscovite-rich (Ms_1_) domains in sample SR10 (crossed polars); **e** rotated Kfs porphyrocryst from sample SR1 within a matrix rich in MS_1_ muscovite (crossed polars); **f** domain rich in dispersed epidote (Ep) (crossed polars); **g** tourmaline (Tur) crystal within a domain rich in muscovite and chloritized (Chl) biotite (Bt; plane polars); **h** tectonic fissure filled by calcite (Cc), cutting the main foliation (sample 104a, crossed polars); **i** macrophotograph of the folded SR25C phyllite sample; **j** microphotograph of the folded mica/chlorite-rich and quartz/feldspar-rich domains in phyllite (sample SR25C, crossed polars); **k** axial part of crenulation with chlorite nest in phyllite (sample SR25C)

**Fig. 3:**
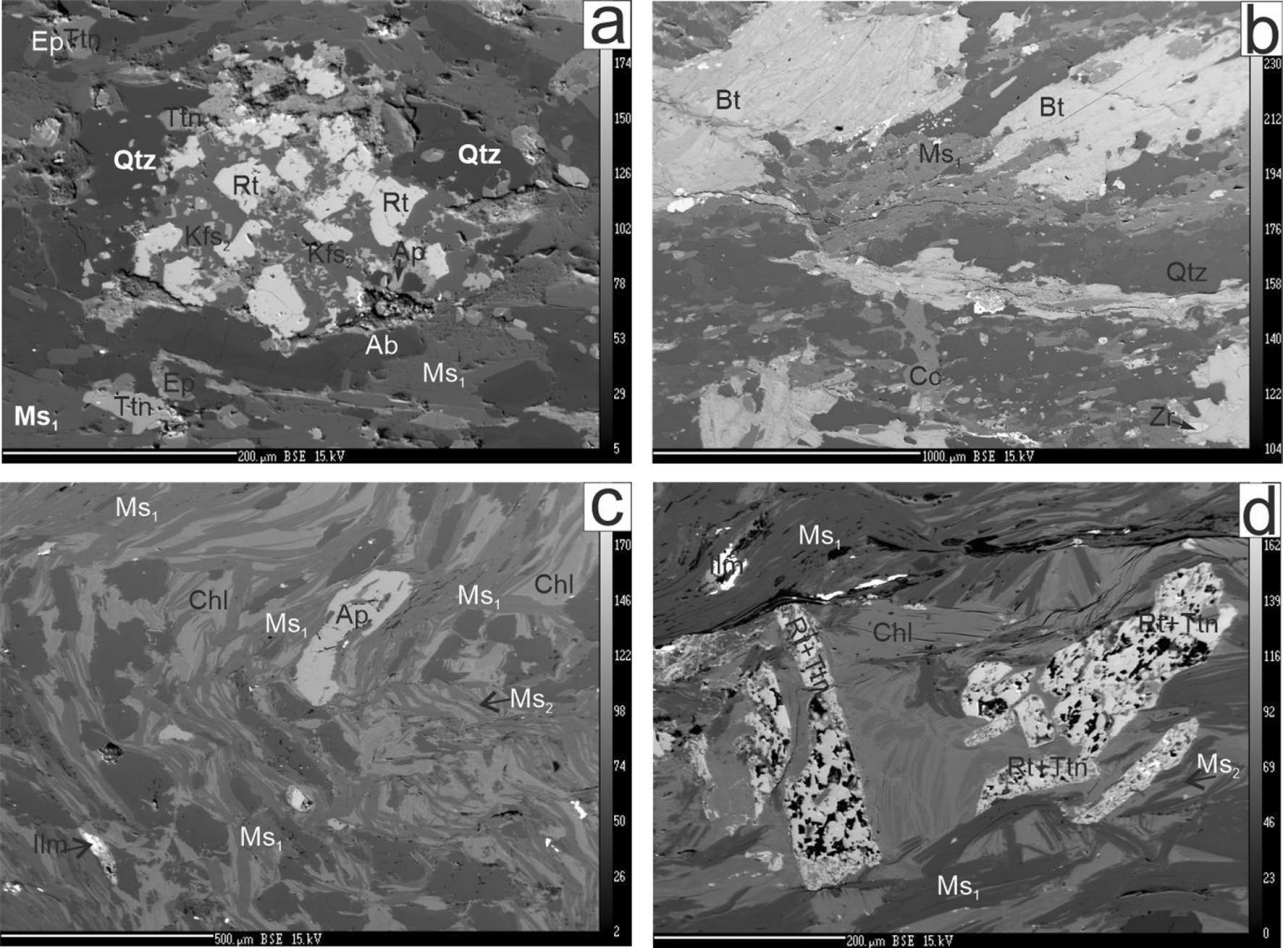
BSE images of the rock textures: **a** aggregate composed of rutile (Rt), titanite (Ttn) and K-feldspar (Kfs), with a foliated matrix of albite (Ab), muscovite (Ms) and titanite (Ttn), SR1 porphyroid; **b** foliation defined by biotite (Bt), muscovite (Ms) and quartz (Qtz), cut by a calcite (Cc) veinlet, 104a porphyroid; **c** strongly crenulated and interleaved chlorite and muscovite with deformed apatite (Ap), SR25C phyllitic meta-tuff; **d** Intergrowths of rutile and titanite (Rt + Ttn) inside a chlorite (Chl) and muscovite domain; note Ms_1_ is discordant to Chl + Ms_2_ intergrowths, SR25C phyllitic meta-tuff

The presence of blue quartz, quartz-feldspar aggregates, K-feldspars (Kfs_1_) and plagioclase porphyroclasts are characteristic features of the porphyroid samples (Fig. 2a, b, d). They are interpreted as remnants of primary phenocrysts that recrystallized during metamorphism. The metamorphic minerals can be grouped into two assemblages, representing two stages of development. The M1 assemblage is composed of biotite exhibiting brick-red to yellow pleochroism (*#fm* = 0.58–0.61; Ti = 0.19–0.24 a.p.f.u.; Table 1), ilmenite, Ms_1_ muscovite rich in Mg and poor in Ti (*mg* = 0.49–0.55 *a.p.f.u*; Ti = 0.02–0.03 a.p.f.u; Table 2), in addition to recrystallized phenocrysts (quartz, Kfs_1_ and plagioclase). The M2 assemblage is represented by Ms_2_ muscovite (*Mg.* = 0.27–0.40 *a.p.f.u* and Ti 0.06–0.07; Table 2), chlorite, titanite, rutile, zoizite (Supplementary Table 1), K-feldspar (Kfs_2_) and tourmaline [dravite_(0.47)_-schorl_(0.20)_-Mg-foitite_(0.12)_-olenite_(0.07)_-ulvite_(0.07)_-foitite_(0.05)_-feruvite_(0.03)_; Supplementary Table 2]. Titanite and rutile form pseudomorphs after ilmenite (Fig. 3a), while epidote, Kfs_2_ and titanite form pseudomorphs after unknown mineral phases and are dispersed throughout the rock (Figs. 2d, 3b). Chlorite (#mg = 0.585–0.570; Table 3) defines bands parallel to the foliation, while rare tourmaline (Fig. 2f) is mostly oriented along the metamorphic lineation. Zircon and apatite are accessory components, while fractures are filled by secondary calcite (Figs. 2g, 3b).

**Table 1:** Chemical composition and crystal-chemical formulae of representative biotite crystals from sample U-104a

Sample No.		U-104a					
Component	LoD	Bt1	Bt2	Bt3	Bt4	Bt5	Bt6
SiO_2_	0.04	34.83	36.00	36.34	36.19	36.99	35.84
TiO_2_	0.06	1.67	1.88	1.99	2.06	1.83	1.66
Al_2_O_3_	0.03	17.14	17.39	17.28	17.04	16.79	17.08
Cr_2_O_3_	0.01	0.34	0.01	0.07	0.05	b.d.l	b.d.l
FeO	0.08	23.43	22.52	22.31	22.27	22.06	22.46
MgO	0.02	8.98	8.45	8.49	8.16	8.64	8.57
MnO	0.02	0.36	0.40	0.40	0.43	0.36	0.33
Na_2_O	0.04	0.11	0.12	0.19	b.d.l	0.05	0.15
K_2_O	0.05	7.54	8.98	9.24	9.41	9.68	9.01
BaO	0.01	0.11	0.19	0.13	0.11	0.05	0.20
Total		94.50	95.93	96.42	95.727	96.449	95.307
		Crystal-chemical formulae recalculated for 22 O^2−^					
Si		5.421	5.526	5.546	5.571	5.639	5.544
Al^iv^		2.579	2.474	2.454	2.429	2.361	2.456
Al^vi^		0.564	0.672	0.654	0.662	0.655	0.658
Ti		0.196	0.217	0.228	0.238	0.21	0.193
Cr		0.041	0.001	0.008	0.007	-	-
Fe		3.05	2.891	2.847	2.867	2.812	2.905
Mg		2.083	1.934	1.931	1.874	1.963	1.976
Mn		0.047	0.052	0.052	0.056	0.047	0.044
Na		0.032	0.036	0.055	-	0.014	0.046
K		1.496	1.758	1.799	1.847	1.883	1.778
Ba		0.007	0.011	0.008	0.007	0.003	0.012
*#mg*		0.067	0.064	0.066	0.062	0.411	0.405
*T*_*H(2005)*_		563	584	594	601	579	559
*T*_*Wu&CHEN2015*_		562	594	601	613	587	573

*b.d.l.* below detection limit, *#mg* Mg/(Mg + Fe); *T*_*H*_ temperature of crystallization (after Henry et al. 2005)

**Table 2:** Chemical composition and crystal-chemical formulae of representative muscovite crystals

Sample No.		104a	104a	104a	SR2	SR2	SR25C	SR25C	SR25C	SR25C	SR25C
Component	LoD	Ms1_1	Ms1-2	Ms2-1	Ms1-1	Ms1-2	Ms1-1	Ms1-2	Ms2-1	Ms2-2	Ms2-3
SiO_2_	0.04	50.85	48.10	47.82	50.10	47.11	49.28	50.29	46.92	45.64	45.54
TiO_2_	0.04	0.25	0.36	0.44	0.34	0.65	0.40	0.34	0.53	0.69	0.79
Al_2_O_3_	0.03	27.96	30.45	31.73	28.31	33.39	29.82	28.24	33.65	35.36	35.91
Cr_2_O_3_	0.01	b.d.l	b.d.l	b.d.l	0.01	0.02	0.01	b.d.l	b.d.l	0.03	0.04
V_2_O_3_	0.01	0.02	0.02	b.d.l	0.05	b.d.l	0.01	b.d.l	0.01	0.04	0.03
MgO	0.02	2.48	2.00	1.70	2.76	1.37	2.37	2.68	1.21	0.55	0.53
FeO	0.10	3.21	3.77	2.73	2.66	1.78	2.72	3.06	1.82	1.13	1.00
MnO	0.02	b.d.l	0.07	b.d.l	b.d.l	b.d.l	b.d.l	b.d.l	0.04	0.06	b.d.l
BaO	0.05	0.32	0.61	0.67	0.17	0.30	0.16	0.20	0.31	0.44	0.50
Na_2_O	0.03	0.19	0.21	0.33	0.39	0.60	0.62	0.44	0.66	0.95	1.05
K_2_O	0.04	10.55	10.57	10.76	10.10	10.29	9.99	9.99	10.31	9.70	9.76
Total		95.83	96.16	96.18	94.89	95.51	95.38	95.24	95.46	94.59	94.10
Crystal-chemical formulae recalculated for 22 O_2_^−^											
Si		6.782	6.453	6.389	6.718	6.279	6.581	6.731	6.262	6.122	6.076
Al^IV^		1.218	1.547	1.611	1.282	1.721	1.419	1.269	1.738	1.878	1.924
Al^VI^		3.177	3.269	3.385	3.192	3.523	3.274	3.185	3.555	3.713	3.723
Ti		0.025	0.036	0.044	0.034	0.066	0.040	0.034	0.053	0.070	0.079
Cr		–	–	–	0.001	0.002	0.001	–	–	0.003	0.004
V		0.003	0.003	–	0.005	–	0.001	–	0.002	0.003	0.004
Mg		0.493	0.400	0.339	0.552	0.273	0.471	0.535	0.241	0.110	0.105
Fe		0.358	0.423	0.305	0.298	0.178	0.304	0.333	0.203	0.127	0.113
Mn		0.001	0.008	0.001	–	–	–	–	0.005	0.007	–
Ba		0.017	0.032	0.035	0.009	0.016	0.008	0.011	0.016	0.023	0.026
Na		0.049	0.056	0.084	0.102	0.154	0.161	0.113	0.172	0.247	0.271
K		1.794	1.809	1.834	1.728	1.749	1.703	1.705	1.756	1.660	1.661
*#mg*		0.579	0.481	0.526	0.649	0.580	0.608	0.616	0.537	0.451	0.482

*b.d.l.* below detection limit, *#mg* Mg/(Mg + Fe)

**Table 3:** Selected chlorite EMP analysis with their chemical formulae based on 28 oxygens and with Fe^2+^/Fe^3+^ and OH calculated assuming full site occupancy

Samples	Sr1					Sr25					104a				
	1	2	3	4	5	6	7	8	9	10	11	12	13	14	15
SiO_2_ (wt%)	26.73	26.24	26.53	25.11	26.71	26.07	25.56	25.23	26.32	25.73	26.78	25.11	26.74	26.51	26.75
TiO_2_	0.05	0.06	0.04	b.d.l	0.07	b.d.l	0.07	b.d.l	0.09	0.14	0.05	0.06	b.d.l	0.06	b.d.l
Al_2_O_3_	19.40	19.07	19.38	16.73	19.49	20.43	22.53	21.92	20.35	21.58	19.53	16.73	19.61	19.18	19.81
Cr_2_O_3_	0.06	0.08	b.d.l	0.10	0.04	b.d.l	b.d.l	b.d.l	b.d.l	b.d.l	b.d.l	b.d.l	0.04	b.d.l	b.d.l
Fe_2_O_3_	0.82	0.26	0.91	b.d.l	0.91	0.92	0.72	0.33	0.96	0.71	0.57	b.d.l	0.63	0.60	0.61
FeO	20.71	22.08	20.49	21.32	20.76	26.84	22.55	23.35	26.95	23.30	21.11	21.81	21.64	21.80	21.75
MnO	0.49	0.44	0.44	0.38	0.46	0.16	0.20	0.24	0.29	0.23	0.50	0.39	0.44	0.39	0.46
MgO	16.74	16.65	16.70	17.12	16.77	13.02	15.70	15.53	12.93	15.25	17.10	16.43	16.93	16.68	16.86
CaO	0.10	0.08	0.07	b.d.l	0.07	b.d.l	b.d.l	b.d.l	b.d.l	0.08	b.d.l	0.06	0.06	b.d.l	0.05
Na_2_O	0.07	0.10	b.d.l	0.09	b.d.l	b.d.l	b.d.l	b.d.l	b.d.l	b.d.l	0.11	0.08	b.d.l	b.d.l	b.d.l
K_2_O	b.d.l	b.d.l	b.d.l	b.d.l	b.d.l	0.00	0.00	0.00	0.05	0.08	b.d.l	0.04	b.d.l	b.d.l	0.04
H_2_O*	11.25	11.17	11.18	10.59	11.27	11.22	11.50	11.34	11.28	11.39	11.33	10.55	11.34	11.21	11.37
Total	96.42	96.24	95.75	91.43	96.56	98.66	99.05	98.03	99.36	98.48	97.07	91.26	97.42	96.45	97.70
Si (apfu)	5.68	5.62	5.68	5.66	5.67	5.56	5.32	5.33	5.58	5.41	5.65	5.69	5.64	5.66	5.63
Al ^iv^	2.32	2.38	2.32	2.34	2.33	2.44	2.68	2.67	2.42	2.59	2.35	2.31	2.36	2.34	2.37
Al ^vi^	2.55	2.45	2.57	2.13	2.56	2.71	2.86	2.79	2.68	2.77	2.53	2.18	2.53	2.49	2.55
Ti	0.01	0.01	0.01	–	0.01	–	0.01	–	0.01	0.02	0.01	0.01	–	0.01	–
Cr	0.01	0.01	–	0.02	0.01	–	–	–	–	0.00	–	–	0.01		–
Fe^3+^	0.13	0.04	0.15	–	0.14	0.15	0.11	0.05	0.15	0.11	0.09	–	0.10	0.10	0.10
Fe^2+^	3.68	3.96	3.67	4.11	3.69	4.79	3.93	4.12	4.78	4.09	3.73	4.20	3.82	3.89	3.83
Mn	0.09	0.08	0.08	0.07	0.08	0.03	0.03	0.04	0.05	0.04	0.09	0.08	0.08	0.07	0.08
Mg	5.30	5.32	5.32	5.76	5.31	4.14	4.87	4.89	4.09	4.78	5.38	5.55	5.33	5.31	5.29
Ni	–	–	–	–	–	–	0.04	–	0.02	–	–	–	–	–	–
Ca	0.02	0.02	0.02	–	0.02	–	–	–	–	0.02	0.00	0.02	0.01	–	0.01
Na	0.06	0.08	–	0.08	–	–	–	0.05	–	–	0.09	0.07	–	–	–
K	–	–	–	–	–	–	–	–	0.02	0.04	–	0.02	–	–	0.02
OH*	16.00	16.00	16.00	16.00	16.00	16.00	16.00	16.00	16.00	16.00	16.00	16.00	16.00	16.00	16.00
**∑**_**Cations**_	35.85	35.98	35.81	36.16	35.82	35.81	35.86	35.95	35.81	35.87	35.92	36.12	35.87	35.88	35.88
*Mg/(Fe* + *Mg)*	0.582	0.571	0.583	0.584	0.581	0.543	0.547	0.540	0.546	0.532	0.585	0.570	0.577	0.571	0.575
T_1_	312	320	312	315	313	331	369	368	328	355	316	310	318	315	320
T_2_	301	310	302	304	303	320	358	357	317	344	305	299	307	304	309
T_3_	295	302	295	297	296	317	336	335	315	327	297	295	299	298	301

*b.d.l.* below detection limit, *T*_*1*_ temperature by Cathelineau and Nieva (1985), *T*_*2*_ temperature by Jowett (1995), *T*_*3*_ temperature by Kranidiotis and MacLean (1987)

In phyllite (SR25C; Fig. 2i, j) the M1 metamorphic assemblage is represented only by Ms_1_ + Ilm + Qtz, while the M2 episode is defined by Ms_2_ + Chl + Ttn + Rt. Ms_1_ muscovite, rich in a phengite component (Table 2) and interleaved with quartz-albite layers, defines the strongly folded foliation (Fig. 2h). Primary ilmenite is replaced by intergrowths of titanite and rutile (Fig. 3d; Table 1). Chlorite (#mg = 0.547–0.542; Table 3) interleaved with Ms_2_ muscovite (Fig. 3c) with a minor phengite component (Table 2) replaces primary biotite and lies along the axial planes of crenulations, probably crystallizing in pressure shadows (Fig. 2i). Deformed apatite (Fig. 3c), zircon and monazite (partly decomposed) are the accessory mineral phases. The chlorite in all investigated samples contains 55–81% of the clinochlore (Mg_5_Al)(Si_3_Al)O_10_(OH) end-member, 11–45% of the chamosite (Fe^2+^_5_Al)(Si_3_Al)O_10_(OH) end-member and up to 5% of the pennite (Mn_5_Al)(Si_3_Al)O_10_(OH) end-member (classification after Bailey 1980) (Table 3).

### Whole rock geochemistry

**Fig. 4:**
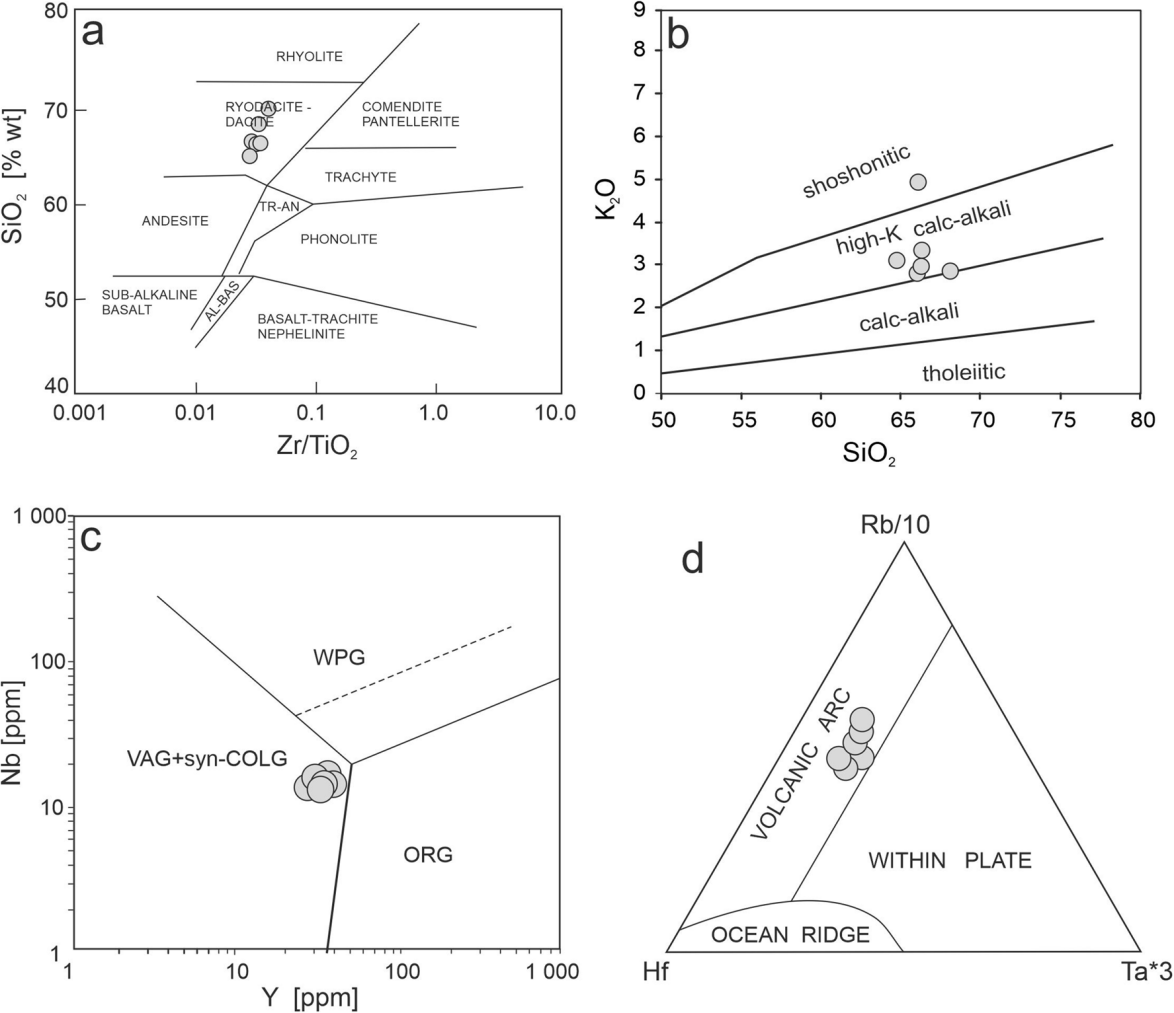
Classification of the porphyroid samples (grey circles) and meta-tuff sample (dark circle) in: **a** classification diagram after Winchester and Floyd (1977); **b** K_2_O versus SiO_2_ classification diagram after Peccerillo and Taylor (1976); **c** discrimination diagram of Pearce et al. (1984): *VAG* volcanic arc granites, *syn-COLG* syn-collisional granites, *WPG* within-plate granites, *ORG* ocean ridge granites; d) discrimination diagram after Harris et al. (1986)

**Fig. 5:**
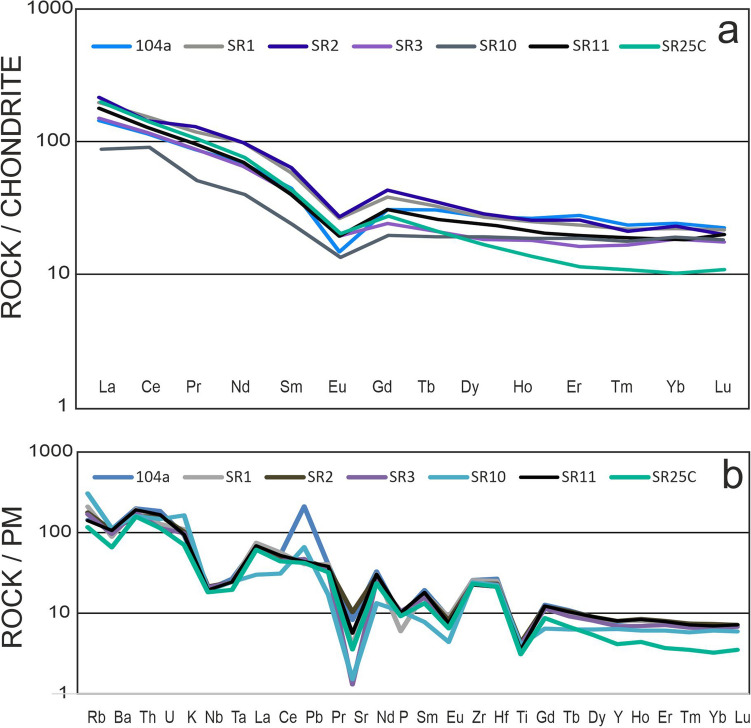
Chondrite (C1) normalized REE patterns (**a**) and primitive-mantle normalized multi-element patterns (**b**) of the analysed porphyroid samples and meta-tuff (SR25C) samples from the Marmarosh Massif. Normalization values after McDonough and Sun (1995)

All samples show a narrow range of SiO_2_ contents (64.72–68.06 wt.%) and are strongly peraluminous in composition (ASI = 1.23–2.82; Table 4). The only elements that vary significantly in concentration between samples are the alkalis, with K_2_O/Na_2_O ratios ranging from 0.8 to 4.25, although their sum is relatively constant (Table 4). The meta-porphyroids as well as the phyllite host rock (interpreted as a meta-tuff; Stepanyuk 1986) plot in the rhyodacite-dacite field on the Winchester and Floyd (1977) discrimination diagram and belong to the high-K calc-alkaline to shoshonitic series with VAG characteristics (Fig. 4a, b). Total REE contents vary from 170 to 234 ppm and their C1-normalized REE patterns are flat with a strongly negative Eu anomaly (Ce_N_/Yb_N_ = 4.75–7.08 for the porphyroids and Ce_N_/Yb_N_ = 12.87 for the phyllitic sample SR25C; Eu/Eu* = 0.40–0.61; Fig. 5a; Table 4).

**Table 4:** Chemical composition and selected petrological indices of the whole-rock porphyroid samples from Dilove Nappe. Marmarosh Massif. Ukraine

Sample No.	LoD	104a	SR1	SR2	SR3	SR10	SR11	SR25C
SiO_2_	0.01	66.00	66.26	64.72	66.24	65.99	68.06	69.65
TiO_2_	0.01	0.83	0.83	0.93	0.87	0.87	0.75	0.67
Al_2_O_3_	0.01	14.90	16.67	14.9	15.13	15.73	14.92	14.08
Fe_2_O_3T_	0.04	5.67	5.7	6.19	6.59	5.37	4.72	4.02
MnO	0.01	0.08	0.6	0.8	0.5	0.06	0.05	0.05
MgO	0.01	2.04	1.7	2.05	2.03	2.2	2.21	1.9
CaO	0.01	2.20	0.26	2.89	0.3	0.29	1.23	0.49
Na_2_O	0.01	3.41	2.29	2.89	3.68	0.16	3.21	3.53
K_2_O	0.01	2.79	3.33	3.09	2.95	4.93	2.84	2.12
P_2_O_5_	0.01	0.20	0.13	0.22	0.23	0.23	0.22	0.2
LOI	–	1.95	2.62	1.46	1.99	3.11	1.98	2.54
Total		100.07	100.39	100.14	100.51	98.94	100.19	99.25
Trace elements [ppm]								
Sr	0.5	173.50	82.6	217.9	27.5	32	119.5	75.1
Ba	1.0	773.00	620	748	673	790	745	460
Rb	0.1	91.20	133.6	113	107.5	194.5	89.7	74.1
Th	0.2	17.00	16.6	13.3	15	13.7	16.3	13.4
U	0.1	3.90	2.7	3.4	2.4	3.1	3.5	2.4
Ga	0.5	19.60	5.1	3.1	3.6	19.7	16.8	15.2
Ni	0.1	19.40	35.1	17.6	17.9	26.2	17.5	23.5
Zr	0.1	290.40	288.5	262.4	264.5	270	251.1	260.1
Hf	0.1	8.30	7.8	7.2	7	6.9	6.5	6.6
Y	0.1	35.40	36.6	36.5	31.5	29	36.5	18.8
Nb	0.1	14.10	15.3	15.3	14.9	13.6	13.8	13.0
Ta	0.1	1.10	1	1	1	1	1	0.8
La	0.1	46.60	51.4	44.2	42.3	20.6	47.2	42.2
Ce	0.1	94.50	101	87	79	55.2	92.3	78.4
Pr	0.02	10.98	11.21	10.05	9.21	4.67	10.51	8.91
Nd	0.30	44.80	41.3	38.6	35.1	18	41.1	31.9
Sm	0.05	8.60	7.77	7.78	6.9	3.45	8.04	5.9
Eu	0.02	1.48	1.52	1.37	1.18	0.74	1.26	1.09
Gd	0.05	7.60	7.16	7.25	6.77	3.84	7.31	5.18
Tb	0.01	1.18	1.08	1.15	0.99	0.68	1.12	0.73
Dy	0.05	6.61	6.34	6.66	5.96	4.64	6.68	3.95
Ho	0.02	1.36	1.32	1.39	1.14	1	1.37	0.72
Er	0.03	3.75	3.64	3.86	3.41	2.93	3.79	1.77
Tm	0.01	0.54	0.55	0.55	0.49	0.43	0.53	0.26
Yb	0.05	3.55	3.47	3.63	3.22	3.01	3.42	1.6
Lu	0.01	0.53	0.53	0.53	0.5	0.44	0.53	0.26
ASI		1.23	2.21	1.16	1.64	2.82	1.48	1.65
Rb/Sr		0.53	1.62	0.52	3.91	6.08	0.75	0.98
Nd/Th		2.64	2.49	2.90	2.34	1.31	2.52	2.38
ΣREE		175.93	232.08	233.87	169.61	225.16	182.87	182.87
Eu/Eu*		0.40	0.56	0.52	0.62	0.50	0.60	0.60
Ce_N_/Yb_N_		4.75	6.99	6.25	6.36	7.09	12.87	12.87
T_Zr_ [ºC]		845	892	823	869	891	852	868

### Zircon petrology and U–Pb data

**Fig. 6:**
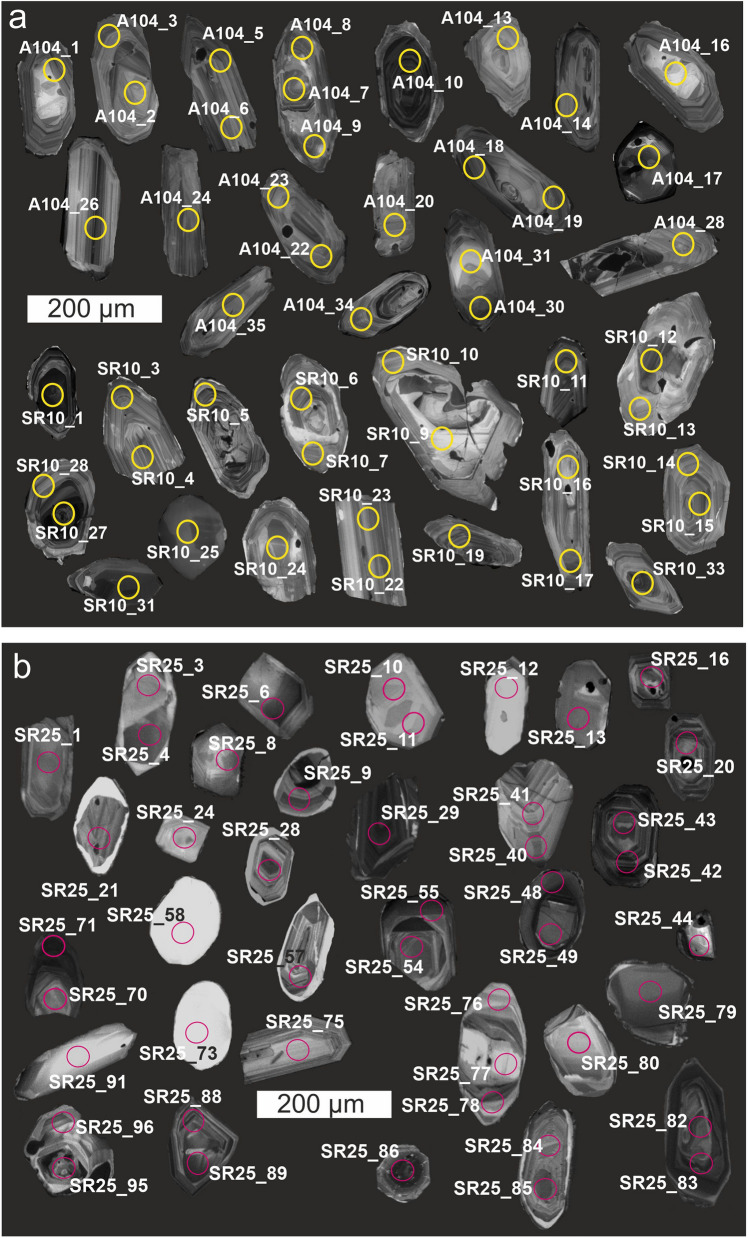
Cathodoluminescence (CL) images of selected (concordant) zircon crystals from (**a**) the meta-volcanic porphyroids (**a**) and (**b**) the meta-tuff (SR25C) from the Marmarosh Massif. Analytical spots are marked as circles

**Fig. 7:**
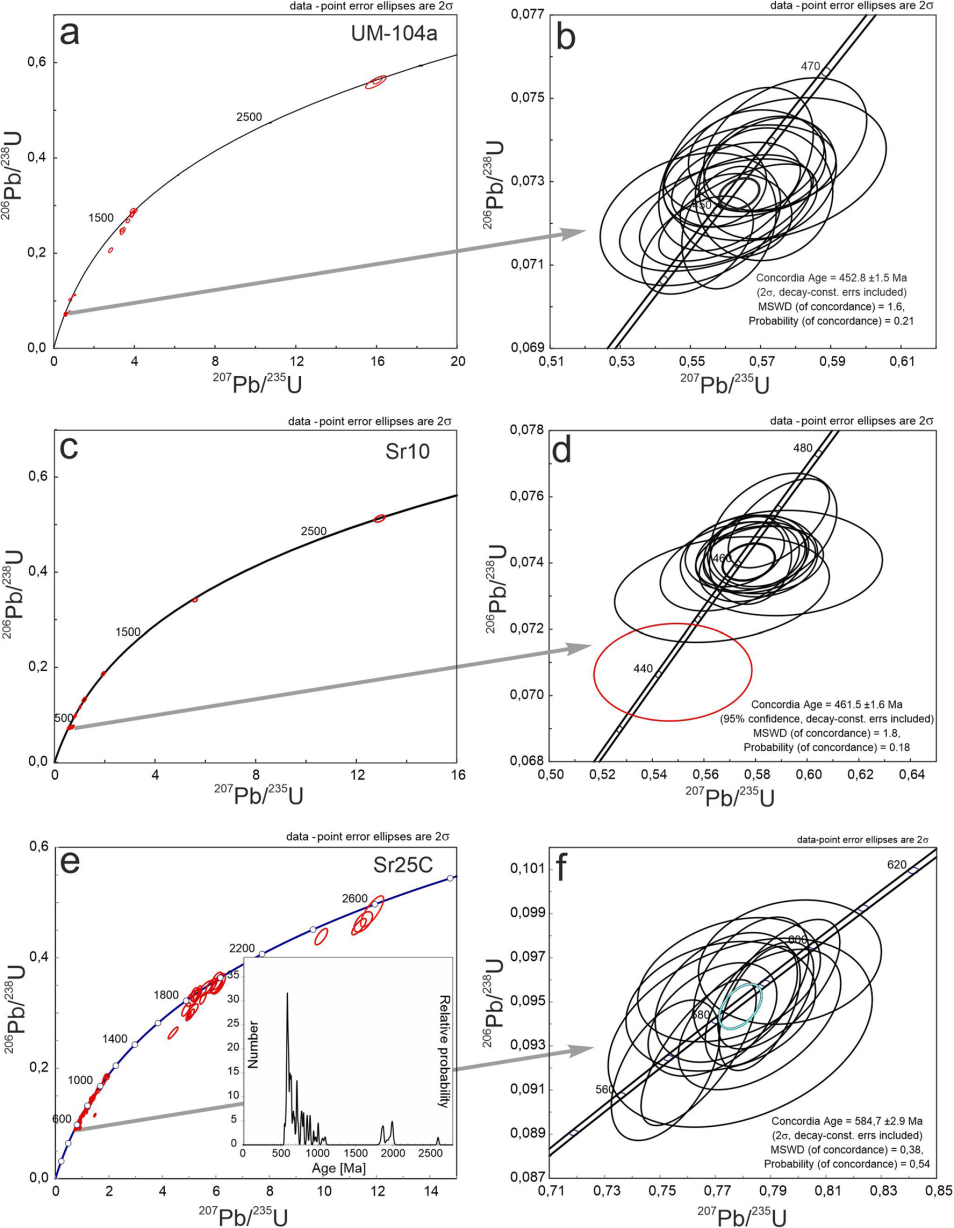
Concordia plots of LA-ICP-MS U–Pb zircon analytical results from the meta-volcanic rocks from Marmarosh Massif: **a** and **b** porphyroid sample 104a; **c** and **d** porphyroid sample SR10; **e** and **f** sample SR25C

Zircon crystals from sample UM-104a and SR-10 are euhedral to subhedral, and short- to long-prismatic (aspect ratios from 3:1 to 1:1). Cathodoluminescence (CL) imaging reveals the presence of magmatic oscillatory zoning, locally overgrowing inherited cores exhibiting both bright and dark CL responses, in both cases with local truncations (Fig. 6a). In sample UM-104a, 38 spot analyses were undertaken on 30 zircon grains. 17 grains with oscillatory zonation yield a concordia age of 452.8 ± 1.5 Ma (MSWD = 1.6, probability of concordance 0.0.21; Supplementary Table 3; Fig. 7a, b). Two inherited cores with oscillatory zoning yielded concordant ages of 624 ± 13 Ma and 673 ± 14 Ma, four inherited cores yielded concordant ages in the 1605–1637 Ma age range and two inherited cores yielded concordant ages of 2879 ± 24 Ma and 2880 ± 10 Ma (Supplementary Table 3; Fig. 7a).

In sample SR10, 34 spot analyses were undertaken on 28 zircon grains. 14 grains showing oscillatory zonation yielded a concordia age of 461.5 ± 1.6 Ma (MSWD = 1.8, probability of concordance = 0.18; Fig. 7c, d). One rim yielded a concordant age of 440.9 ± 6.7 Ma (Supplementary Table 3). Eight inherited cores with oscillatory internal zoning yielded concordant to slightly discordant ages ranging from 585 to 1100 Ma. One inherited core yielded a concordia age of 2675 Ma (Supplementary Table 3). Ten discordant analyses were obtained both from the rims and the cores (Fig. 7c).

Zircon crystals from sample SR25C are translucent, euhedral, short- to long-prismatic (aspect ratios from 2:1 to 4:1) and are 80 to 300 μm long. Most of the crystals show magmatic zoning in CL and 96 spot analyses were undertaken on 64 crystals. 16 analyses were discordant (Fig. 6b; Supplementary Table 4). Inherited cores with a weak CL response yielded a broad spectrum of ages: 2602 Ma, 1991–1920 Ma, four cores which together yield a concordia age of 1862 ± 11 Ma and six cores which yielded ages of 1093–940 Ma. 48 oscillatory zoned crystals, showing moderate CL response yielded a broad spectrum of concordant Neoproterozoic ages from 893 to 596 Ma, (including nine crystals with a concordia age of 636.1 ± 4.0 Ma; Fig. 7e). 13 oscillatory zoned rims with a bright CL response yielded a concordia age of 584.7 ± 2.9 Ma (Fig. 7f), while two rims yielded ages 550.6 and 560.4 Ma (Supplementary Table 4).

## Discussion

### Metamorphic conditions

**Fig. 8:**
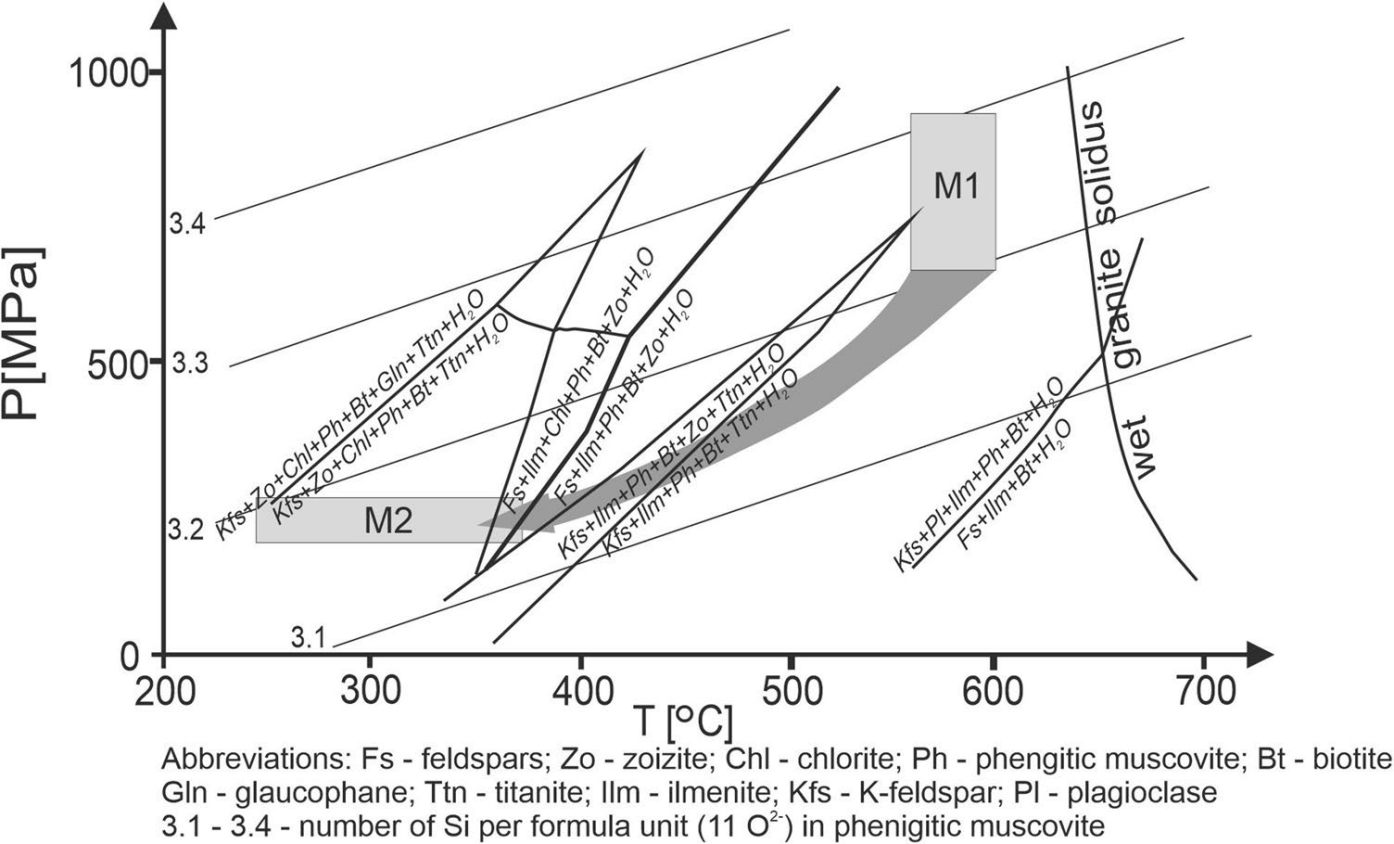
P–T path of the metamorphic evolution of the metavolcanic rocks from the Marmarosh Massif with the stability fields of the main mineral assemblages indicated. See the main text for discussion

Peak metamorphic temperature estimates in the porphyroid samples were undertaken using Ti-in-biotite geothermometry (Henry et al. 2005; Wu and Chen 2015) and are in the c. 560 – 600 °C range (Table 1). The amount of phengite substitution in Ms_1_ muscovite (Table 2), coexisting with K-feldspar (Kfs_1_) and quartz, was used to constrain the peak metamorphic pressure (Massone and Schreyer 1987). Pressure estimates based on the muscovite (Ms_1_) chemistry are c. 600–900 MPa (M1; Fig. 8). In the case of the phyllite sample (SR25C), only phengite-based barometry can be applied, due to a lack of other mineral phase suitable for geothermometry and yields pressure estimates of 700–900 MPa (Fig. 8).

In the porphyroid samples the low phengite component both in the rims of muscovite and in muscovite interleaving with chlorite (Ms_2_) in pseudomorphs after biotite implies a pressure of c. 200–250 MPa. Ternary feldspar geothermometry (Fuhrman and Lindsley 1988) yielded a temperature range of 222–384 °C (Table 5).

**Table 5:** Modal composition and temperature estimate obtained using the Fuhrman and Lindsley (1988) ternary feldspars geothermometry for feldspars from the exotic block from Pluskawka microgranite (Pl 2)

Feldspar type/composition	Ab	Or	An
K-feldspar and plagioclase			
Plagioclase Original/odjusted	0.936/0.975	0.001/0.024	0.010/0.009
Alkali feldspar Original/adjusted	0.034/0.047	0.932/0.953	0.002/0.000
Concordant temperature [°C]	371.07	282.11	371.03
Average temperature [°C]	341.40		
Plagioclase Original/odjusted	0.964/0.955	0.016./0.012	0.012/0.034
Alkali feldspar Original/adjusted	0.023/0.006	0.973/0.994	0.002/0.000
Concordant temperature [°C]	221.71	221.71	221.71
Average temperature [°C]	221.71		
Plagioclase Original/odjusted	0.968/0.943	0.010/0.033	0.032/0.024
Alkali feldspar Original/adjusted	0.034/0.064	0.946/0.936	0.010/0.000
Concordant temperature [°C]	403.98	344.04	403.98
Average temperature [°C]	384.00		

In addition to standard geothermometry and geobarometry, a stable phase diagram for porphyroid (U104a sample) was constructed using the THERIAK DOMINO software (de Capitani and Brown 1987; de Capitani and Petrakakis 2010) in an NCKFMASH system under fully water-saturated conditions. The THERMOCALC database (Powell et al. 1998) and the November 2004 (ds55) update of the Holland and Powell (1998) dataset were used. The following phases and a–X models were implemented: feldspar (Baldwin et al. 2005), biotite, garnet, ilmenite, spinel and melt (White et al. 2007), amphibole, cordierite, and epidote (Holland and Powell 1998), clinopyroxene (Holland and Powell 1996), chlorite (Holland et al. 1998) and white mica (Coggon and Holland 2002). Quartz, titanite, sillimanite, andalusite, kyanite, and water were treated as pure phases. The Ms_2_ + zoizite + titanite + rutile + K-feldspar (Kfs_2_) assemblage implies the presence of a fluid with high oxygen fugacity and high Ca activity (Broska et al. 2007) during M2 metamorphism (Fig. 8). In the same P–T range ilmenite breakdown to titanite started, however, titanite formation replacing ilmenite was also possible at higher temperatures and pressures on M1 → M2 retrograde path (Fig. 8). Ep + Kfs_2_ + Ttn + Rt pseudomorphs (Fig. 3a) could be interpreted as a result of amphibole or pyroxene breakdown during retrogression.

Chlorite flakes are aligned along the foliation in the porphyroids while in the phyllite sample SR25C chlorites and Ms_2_ muscovite define the foliation and crystallized in the axial planes of crenulations (Figs. 2k, 3c). The temperature of chlorite growth was calculated using three various empirical chlorite geothermometers proposed by Cathelineau and Nieva (1985), Kranidiotis and MacLean (1987), and Jowett (1991), which are well suited to constraining chlorite temperatures in various geological environments (e.g. Janeczek et al. 2020; Szopa et al. 2020). The temperatures obtained by the various chlorite geothermometers in the porphyroids range from 280 to 360 °C (Table 3), which is in agreement with the phase diagram constraints (Fig. 8). In the phyllite sample SR25C, a temperature of c. 335 °C was determined. In all investigated samples the chlorite geothermometry estimates together with pressure estimates from the low degree of phengite substitution in Ms_2_ muscovite (Table 2) define the conditions of M2 retrogression (Fig. 8). This retrogression episode can be correlated with the Romanian part of the Marmarosh Massif and in the other parts of the Eastern Carpathians, and is related to Alpine nappe staking and shearing (Munteanu and Tatu, 2003). It has been dated at c. 95 Ma by ^40^Ar/^39^Ar geochronology (Culshaw et al. 2012) in the neighbouring Rodna Mountains (Fig. 1b). No direct dating of this retrogressive metamorphic episode in the Ukrainian Carpathians has been undertaken, but as there is a continuation in the nappe structure we assume a similar origin and timing for M2 retrogression (Fig. 8), marked by crystallization of Ms_2_ muscovite, chlorite and rare tourmaline defining the lineation.

### Protoliths of the porphyroid and phyllite samples

The porphyroids can be interpreted as acid, peraluminous metavolcanics (rhyodacite-dacite; Fig. 4a). The magmatic protolith, with a high K-calc-alkaline to shoshonitic chemistry (Fig. 4b), is likely associated with an arc-related tectonic setting (Fig. 4c, d), which is supported by the negative Ta and Nb anomalies shown on a primitive mantle–normalized multielement plot (Fig. 5b). The negative Ti and slightly positive Th anomalies can imply a crustal component for of the parent magma. Negative Eu anomalies (Fig. 5a; Table 4) suggest the fractional crystallization of the rhyodacite-dacite magmas. The zircon saturation temperatures (after Watson and Harrison 1983) vary from 823 to 892 °C (Table 4). Due to the presence of inherited zircon the magma temperature may be slightly overestimated. The inherited zircon component also supports the presence of a crustal component in the early Paleozoic magmas, with the majority of the inherited cores being Neoproterozoic in age, similar to those found in the host meta-tuff (SR25C), along with some Mesoproterozoic and Archean inherited zircon cores (Supplementary Tables 3 and 4). The porphyroids are dated in this study at 453 and 461 Ma (Fig. 7b, d; Supplementary Table 3) and partly overlap in age with similar meta-igneous bodies dated in the Romanian segment of the Eastern Carpathians (Balintoni and Balica 2013 and references therein), including the neighbouring Rodna Mountains metagranitoids (468 – 465 Ma; Panã et al. 2002) as well as with rhyodacite metavolcanics in the southern Gemericum basement of the Central Western Carpathians (466–464 Ma; Vozárová et al. 2010).

Phyllite sample SR25C shares similar geochemical signatures and consequently can be interpreted as VAG-related rhyodacite-dacite, calc-alkaline to high-K calc-alkaline meta-tuff (Fig. 4a–c). The Zr saturation temperature was calculated at 868 °C (Table 4). However, the youngest zircon population exhibits typical magmatic zoning (Fig. 6b) and yielded a concordia age of 584.7 ± 2.9 Ma with two outermost rims dated at c. 550 and c. 560 Ma (Fig. 7d). The broad spectrum of older zircon ages implies significant incorporation of inherited or detrital zircon. The sample is interpreted as a Neoproterozoic tuff with a volcanic-arc protolith, with negative Eu anomalies on C1-normalized REE whole rock diagrams (Fig. 5a). The U–Pb zircon concordia age of 584.7 ± 2.9 Ma is interpreted as the maximum estimate for the eruption age.

### Geotectonic significance of the data

The Ordovician magmatic arc setting, together with the dominance of Neoproterozoic inherited components, allow us to speculate on possible tectonic scenarios (primarily a Baltican *vs* Gondwanan affinity).

In the first scenario, the arc is interpreted as a terrane of a peri-Gondwanan affinity (*cf* Balintoni and Balica 2013; Balintoni et al. 2014). These authors, working in the Romanian Carpathians, suggested the magmatic arc developed on the margins of Gondwana as a back-arc basin, and subsequently docked onto Gondwana in the Early Ordovician. A similar interpretation of back-arc basin closure as a result of Cenerian (early Palaeozoic; Zurbriggen 2017) orogenic events along the eastern margin of Gondwana has been applied to many pre-Alpine crystalline complexes in the Alps (Zurbriggen 2017 and references therein) and Central Western Carpathians (Putiš et al. 2008; Vozárová et al. 2010; Burda et al. 2021). In such a scenario, the Marmarosh Massif, as well as the whole Bucovinian nappe, can be linked to the Central Western Carpathians and Alps. Balintoni and Balica (2013) assumed that Nd T_DM_ model ages of c. 2.0 Ga in the arc-related magmatic rocks imply an east (peri-) Gondwanan provenance. However, the southern portions of Baltica (i.e. Sarmatia), also show similar c. 2.0 Ga mantle extraction ages (Bogdanova et al. 2015 and references therein) and hence could also represent the melt source, as discussed below.

**Fig. 9:**
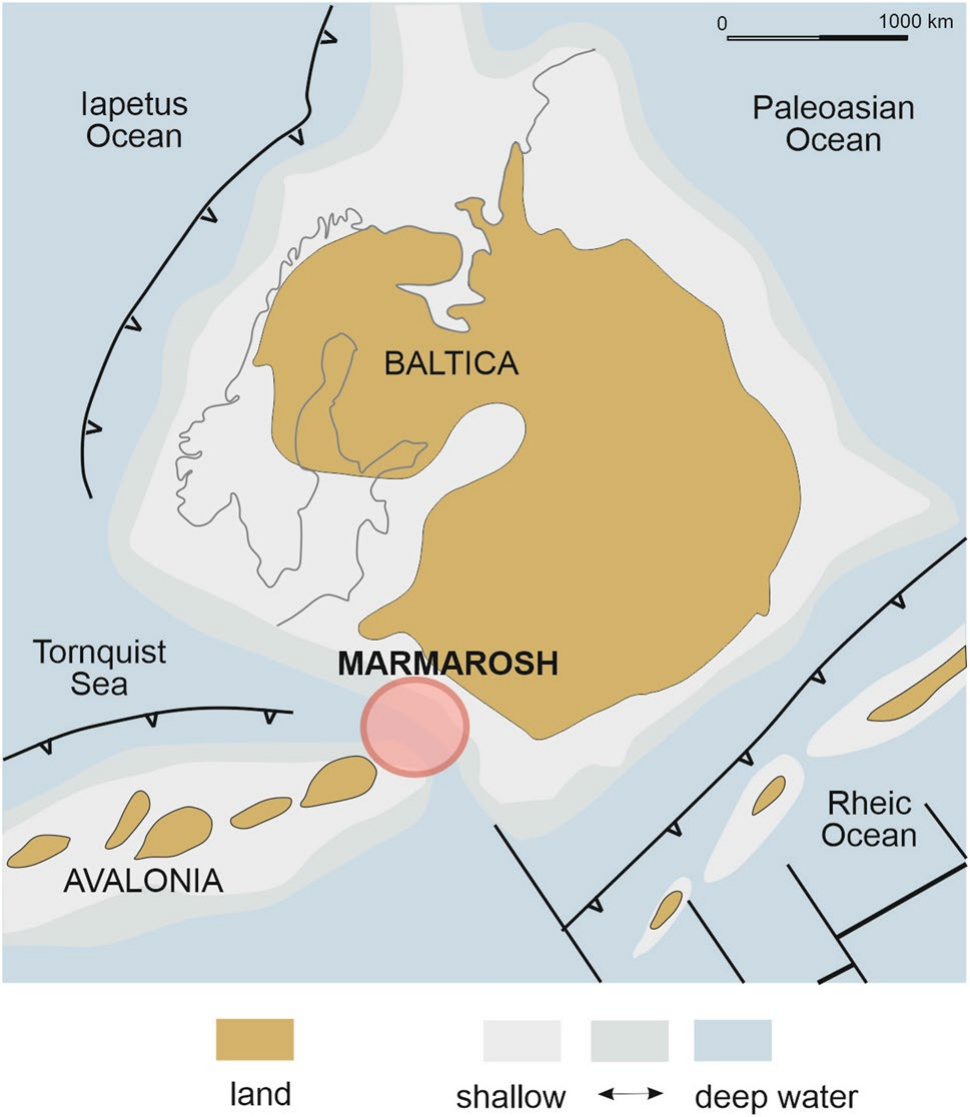
Palaeogeographic map of Baltica, Eastern Avalonia and adjacent areas during the Late Ordovician (c. 452 Ma). Modified after Golonka et al. (2019)

The second scenario assumes the pre-Alpine Eastern Carpathian terranes represent the eastern prolongation of East Avalonia arc terranes related to the closure of the Tornquist Ocean (Munteanu and Tatu 2003; Balintoni et al. 2010; Gawęda and Golonka 2011) (Fig. 9). This eastern branch of the Caledonian orogen has been a matter of debate since the first half of the twentieth century (see Kossmat 1936; Kõberl 1959). In Central Europe, Avalonia-Baltica convergence is assumed to represent a “zipper” tectonics style (progressive closure of an ocean from one end to the other), with Baltica being progressively thrust under Avalonia due to the “soft” closure of the Tornquist Ocean (e. g. Mazur et al. 2018 and references therein). In this scenario, the eastern edge of the Tornquist Ocean can be interpreted as an active continental margin during Caledonian orogenic events, with the magmatic arc containing remnants of Neoproterozoic, Mesoproterozoic and Archean crust (Supplementary Table 3; Fig. 7a, c) and docking to Baltica during Caledonian times. In this model the Nd T_DM_ model ages of c. 2.0 Ga discussed by Balintoni and Balica (2013) can be interpreted as inherited from a Sarmatian-type crustal component, typical of many Avalonian terranes (see Thompson et al. 2012 and references therein for comparison).

The key to resolving differences between these two scenarios is the Variscan and Alpine history of the Marmarosh Massif. During the Alpine orogeny, the Marmarosh Massif was a part of the North European Platform, which rifted away during the opening of the Carpathian branch of Tethys (Protosilesian-Severin-Moldavidic Basin) (Schmidt et al. 2008; Golonka et al. 2021a and references therein). In the first scenario, with a magmatic arc developing along the east Gondwana margin, the question thus arises when did the Marmarosh Massif dock to Baltica/Laurussia? If it was not during the Caledonian orogenesis, the only other plausible timing for docking is during the Variscan cycle, which is considered a hot and magma-rich orogenic episode (see Franke et al. 2021 and references therein). However, both in the Ukrainian and Romanian portions of the Marmarosh Massif no Variscan granitoids/volcanics have been found. The late Variscan (c. 310 Ma Rb–Sr whole-rock dates; Krautner 1991 and references therein; and c. 318 Ma CHIME monazite dates; Reiser et al. 2019) or Permian–Triassic (280–200 Ma on the basis of ^40^Ar/^39^Ar laser single-grain dating; Culshaw et al. 2012) ages for greenschist-facies metamorphism in the Marmarosh Massif may be the result of distant Variscan orogenic events (e.g. overthrusting and nappe formation, Mazur et al., 2020) or post-Variscan, pre-Cimmerian extension, basaltic underplating and the formation of the Meliata Ocean (Stüve and Schuster, 2010; Culshaw et al., 2012). All these facts thus support the second scenario, with an Avalonian provenance for the Marmarosh Massif (Fig. 9) which docked with Laurussia during the Caledonian orogeny.

## Conclusions

1. The porphyroid rocks of the Dilove Nappe in the Marmarosh Massif represent an Ordovician volcanic arc-related rhyodacite sequence, dated at 452.8 ± 1.5 Ma and 461.5 ± 1.6 Ma. The data imply the volcanic arc represents early Caledonian igneous activity and subsequent closure of the easternmost Tornquist Ocean on the margin of Baltica rather than Cenerian orogenic events on the margin of East Gondwana.
2. The Neoproterozoic country rocks are meta-tuffs with a maximum estimate for the eruption of 584.7 ± 2.9 Ma.
3. The petrochronological data allow also a correlation of the Dilove Nappe with the Bucovinia Nappe of the Romanian part of Marmarosh Massif. Peak (M1) metamorphism took place at pressures of 600–900 MPa and temperatures of 560–600 °C. The (M2) retrogression, possibly related to Alpine nappe stacking and shearing, took place at pressures of 250–200 MPa and temperatures of 384–222 °C.

## Supplementary Information

Below is the link to the electronic supplementary material.

Supplementary file1 (DOCX 50 KB)

Supplementary file2 (DOCX 16 KB)

Supplementary file3 (XLSX 26 KB)

Supplementary file4 (XLSX 31 KB)
