# Supplementary material for: Collision with Gondwana or with Baltica? Ordovician magmatic arc volcanism in the Marmarosh Massif (Eastern Carpathians, Ukraine)
Source: Int J Earth Sci. 2022 Jul 16;111(7):2181–98. doi: 10.1007/s00531-022-02228-8 (PMC9464182; doi:10.1007/s00531-022-02228-8)
Supplement: Supplementary file 1 — Supplementary file1 (DOCX 50 KB) [file 531_2022_2228_MOESM1_ESM.docx]

Supplementary Table 1

Chemical composition and crystal-chemical formulae of titanite, zoizite and rutile from porphyroids (U104a) and phyllites (SR25C)

| Compound (wt%) | LoD | Zoizite | | | Titanite | | Rutile | |
| --- | --- | --- | --- | --- | --- | --- | --- | --- |
|  |  | U104_1 | U104_2 | SR25C_1 | U104_1 | U104_2 | SR25C_1 | SR25C_2 |
| V_2_O_5_ | 0.10 | - | - | - | 0.12 | 0.14 | b.d.l. | 0.11 |
| Nb_2_O_5_ | 0.05 | - | - | - | 0.10 | 0.09 | b.d.l. | 0.09 |
| SiO_2_ | 0.05 | 38.85 | 38.51 | 37.92 | 31.64 | 30.45 | 0.06 | 0.14 |
| TiO_2_ | 0.03 | 0.18 | 0.10 | 0.13 | 33.72 | 32.56 | 97.31 | 97.14 |
| Cr_2_O_3_ | 0.02 | 0.04 | 0,05 | b.d.l. | 0.04 | b.d.l. | b.d.l. | b.d.l. |
| Y_2_O_3_ | 0.06 | b.d.l. | b.d.l. | b.d.l | 0.75 | 1.19 | b.d.l. | b.d.l. |
| Al_2_O_3_ | 0.01 | 28.73 | 27.51 | 25.61 | 4.42 | 5.29 | 0.03 | 0.04 |
| Fe_2_O_3_ | 0.07 | 6.13 | 7.40 | 10.81 | 0.62 | 0.43 | 2.50 | 1.50 |
| CaO | 0.03 | 23.98 | 23.99 | 23.34 | 27.08 | 28.26 | - | - |
| MnO | 0.05 | 0.10 | 0.17 | 0.28 | 0.06 | b.d.l. | b.d.l. | b.d.l. |
| H_2_O_calc_ | - | 1.94 | 1.92 | 1.91 | ---- | ---- | - | - |
| F | 0.03 | - | - | - | 1.39 | 2.21 | - | - |
| O=F |  |  |  |  | 0.58 | 0.93 | - | - |
| **Total** | |  |  |  |  |  |  |  |
|  | | formula based on 13 O^2-^ | | | formula based on 3 cations | | formula based on 2 O^2-^ | |
| V^5+^ | | - | - | - | 0.002 | 0.002 | 0.001 | 0.001 |
| Nb^5+^ | | - | - | - | 0.001 | 0.001 | - | 0.001 |
| Si^4+^ | | 2.988 | 2.980 | 2.984 | 0.997 | 1.000 | 0.001 | 0.002 |
| Ti^4+^ | | 0.012 | 0.010 | 0.007 | 0.819 | 0.786 | 0.974 | 0.983 |
| Cr^3+^ | |  |  |  | 0.001 | - | - | - |
| Y^3+^ | |  |  |  | 0.012 | 0.021 | - | - |
| Al^3+^ | | 2.716 | 2.531 | 2.376 | 0.168 | 0.184 | 0.001 | 0.001 |
| Fe^3+^ | | 0.357 | 0.434 | 0.641 | 0.015 | 0.011 | 0.025 | 0.015 |
| Ca^2+^ | | 1.988 | 2.000 | 1.968 | 0.922 | 0.999 | - | - |
| Mn^2+^ | | 0.002 | 0.011 | 0.019 | 0.002 | - | - | - |

Abbreviations: b.d.l. – below detection limit.
