# Supplementary material for: Collision with Gondwana or with Baltica? Ordovician magmatic arc volcanism in the Marmarosh Massif (Eastern Carpathians, Ukraine)
Source: Int J Earth Sci. 2022 Jul 16;111(7):2181–98. doi: 10.1007/s00531-022-02228-8 (PMC9464182; doi:10.1007/s00531-022-02228-8)
Supplement: Supplementary file 2 — Supplementary file2 (DOCX 16 KB) [file 531_2022_2228_MOESM2_ESM.docx]

Table 5. Microchemical analyses and the crystal-chemical chemistry of selected tourmaline crystals from U-104a sample.

| **Compound** | **Tr#1** | **Tr#2** | **Tr#3** | **Tr#4** | **Tr#5** | **Tr#6** |
| --- | --- | --- | --- | --- | --- | --- |
| SiO_2_ | 37.21 | 37.17 | 37.03 | 37.47 | 36.87 | 37.10 |
| TiO_2_ | 0.74 | 0.80 | 0.77 | 0.76 | 0.63 | 0.71 |
| Al_2_O_3_ | 32.78 | 32.78 | 32.65 | 33.67 | 33.06 | 32.83 |
| FeO | 5.12 | 5.22 | 5.27 | 6.00 | 5.24 | 5.10 |
| MnO | b.d.l. | b.d.l. | b.d.l. | 0.05 | 0.07 | b.d.l. |
| MgO | 7.50 | 7.51 | 7.53 | 6.48 | 7.47 | 7.44 |
| CaO | 0.53 | 0.56 | 0.55 | 0.30 | 0.49 | 0.46 |
| Na_2_O | 2.32 | 2.33 | 2.19 | 2.16 | 2.23 | 2.22 |
| K_2_O | 0.00 | 0.01 | 0.00 | 0.03 | 0.00 | 0.00 |
| B_2_O_3_* | 9.27 | 10.25 | 10.73 | 9.35 | 10.79 | 10.23 |
| H_2_O | 3.15 | 3.15 | 3.15 | 3.15 | 3.15 | 3.15 |
| F | 1.40 | 0.24 | 0.12 | 0.60 | b.d.l. | 0.74 |
| SUMA | 100.00 | 100.01 | 100.00 | 100.03 | 100.00 | 100.00 |
| O=F | -0.59 | -0.10 | -0.05 | -0.25 | - | -0.31 |
| **Total** | **99.41** | **99.91** | **99.95** | **99.78** | **100.00** | **99.69** |
| atoms per formula unit per 31 O2^-^ and permanent water content 3.15 wt%. | | | | | | |
| Si^4+^ | 6.12 | 6.09 | 6.05 | 6.16 | 6.02 | 6.07 |
| Ti^4+^ | 0.09 | 0.10 | 0.09 | 0.09 | 0.08 | 0.09 |
| Al^3+^ | 6.36 | 6.33 | 6.29 | 6.53 | 6.36 | 6.33 |
| Fe^2+^ | 0.70 | 0.72 | 0.72 | 0.82 | 0.72 | 0.70 |
| Mn^2+^ | - | - | - | 0.01 | 0.01 | - |
| Mg^2+^ | 1.84 | 1.83 | 1.83 | 1.59 | 1.82 | 1.81 |
| Ca^2+^ | 0.09 | 0.10 | 0.10 | 0.05 | 0.09 | 0.08 |
| Na^+^ | 0.74 | 0.74 | 0.69 | 0.69 | 0.70 | 0.71 |
| K+ | - | - | - | 0.01 | - | - |
| B^3+^ | 2.63 | 2.90 | 3.02 | 2.66 | 3.04 | 2.89 |
| OH^-^ | 3.46 | 3.44 | 3.43 | 3.46 | 3.43 | 3.44 |
| F^-^ | 0.73 | 0.12 | 0.06 | 0.31 | - | 0.38 |

* Boron content calculated assuming full site occupancy.

Abreviations: b.d.l. – below detection limit
